# Case report: A rare case of chondrosarcoma-like malignant giant cell tumor in adolescent rib: diagnostic challenges and treatment

**DOI:** 10.3389/fonc.2024.1523104

**Published:** 2025-01-15

**Authors:** Zhuolin Qin, Longqian Li, Tao Jing, Cheng Wang

**Affiliations:** ^1^ The Second Clinical Medical College, Lanzhou University, Lanzhou, China; ^2^ Department of Thoracic Surgery, Lanzhou University Second Hospital, Lanzhou, Gansu, China

**Keywords:** case report, giant cell tumors, malignant, rib, chondrosarcoma-like

## Abstract

Chondrosarcoma-like malignant giant cell tumor (GCT) of the rib is an extremely rare and aggressive tumor, particularly in adolescents. This case report describes a 19-year-old female presenting with a GCT of the rib with chondrosarcomatous differentiation, highlighting the challenges posed by its unusual location and pathological complexity. Multidisciplinary diagnostic approaches, including advanced imaging, immunohistochemistry (IHC), and pathology, were essential for confirming the diagnosis. Key IHC markers such as Vimentin, SMA, and CD163, alongside genetic analysis excluding H3F3A mutations, guided the diagnostic process. The patient underwent successful surgical resection, achieving early recovery without adjuvant therapy. This report underscores the importance of early detection, precise pathological evaluation, and individualized surgical treatment for rare and high-risk tumors, emphasizing the need for long-term follow-up to monitor recurrence.

## 1 Introduction

Primary malignant bone tumors of the rib are extremely rare in clinical practice, especially among adolescents, with limited cases reported in the literature (1). Giant cell tumor (GCT) typically arise in the epiphyseal regions of long bones, such as the distal femur, proximal tibia, and distal radius, predominantly affecting individuals aged 20 to 40 (2). Although these long bones are typical sites of GCT occurrence, GCT in the rib is exceptionally rare (3). According to the WHO Classification of Tumors in Soft Tissue and Bone (5th edition, 2020), chondrosarcomatous differentiation in GCTs represents a distinct pathological entity with unique clinical and prognostic implications (4). Rib GCTs in adolescents are even more uncommon, often presenting as localized pain and swelling—symptoms that are easily misdiagnosed as benign conditions, such as costochondritis, rib fractures, or other non-specific inflammations (5).

These atypical presentations require clinicians to maintain high vigilance during the diagnostic process, with comprehensive imaging evaluations to exclude potential malignancies (6). In rare cases where GCTs show chondrosarcomatous differentiation, diagnosis and treatment become even more complex (7). This differentiation suggests a higher potential for malignancy and aggressiveness, possibly accompanied by a greater risk of recurrence (8). Such cases are rarely documented, particularly among adolescents with rib involvement.

Given the complexity and potential for high recurrence of such tumors, early and accurate diagnosis is critical (9). A multidisciplinary diagnostic and treatment strategy, including thorough clinical evaluation, imaging studies, pathological analysis, and necessary genetic testing, plays a key role in managing these rare tumors (10). Through detailed case reporting and analysis, we aim to provide a more comprehensive understanding and reference for the diagnosis and treatment of such rare cases.

## 2 Case report

**Figure 1:**
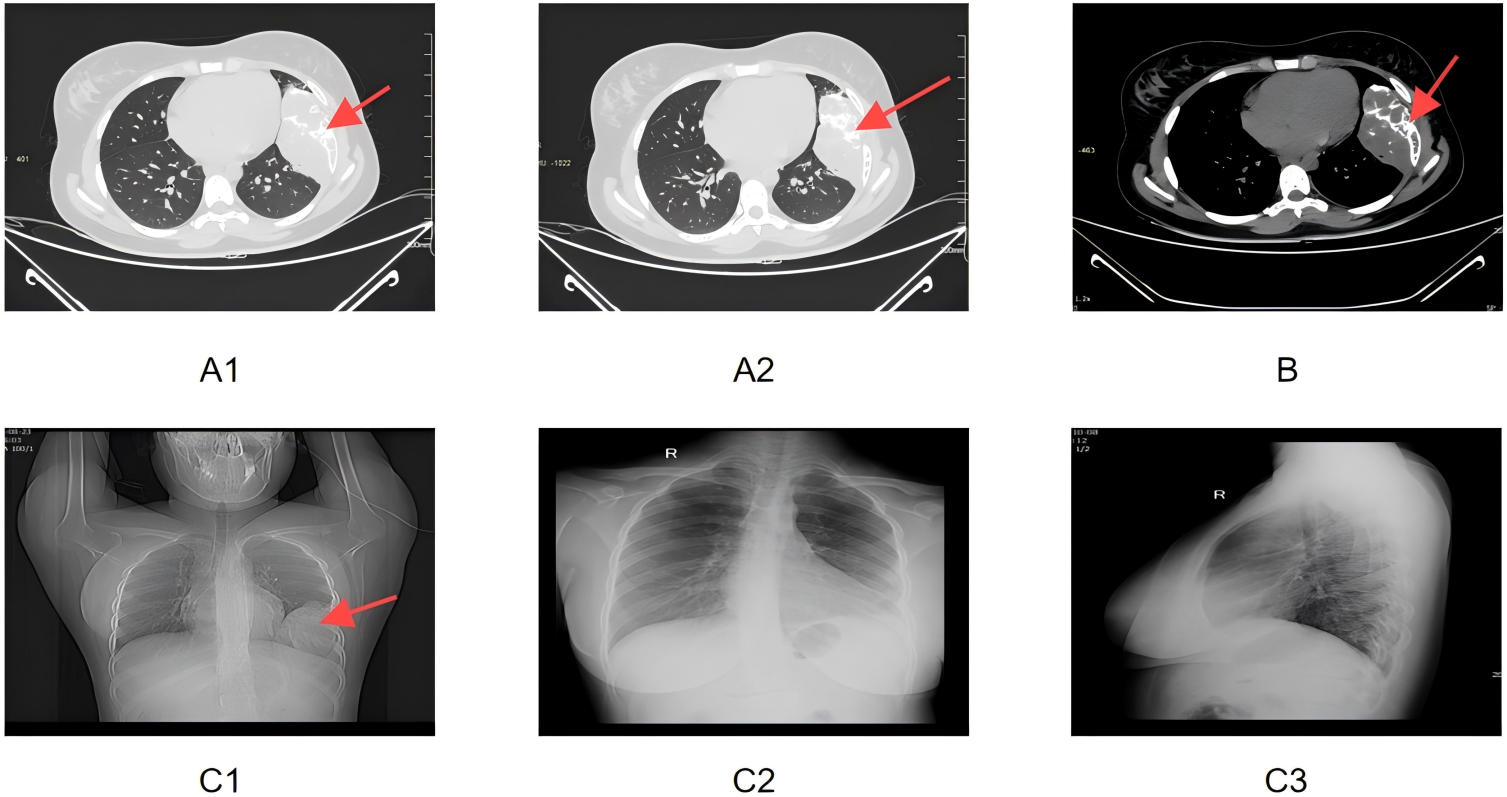
**(A1, A2)** Enhanced CT lung window images showing the expansile, osteolytic destruction of the left 6th rib, accompanied by soft tissue mass formation. Multiple patchy and arc-like calcifications and internal septations are visible. The red arrows point to the area of the rib destruction and the soft tissue mass. **(B)** Enhanced CT mediastinal window image demonstrating destruction of the left 6th rib and associated soft tissue mass, with heterogeneous enhancement. The red arrow highlights the region of the rib damage and the mass’s heterogeneity. **(C1)** Preoperative bedside frontal chest X-ray showing symmetrical thoracic structures, centered trachea and mediastinum, and expansile destruction of the left 6th rib, with soft tissue swelling and increased lung markings. The red arrow indicates the expansile destruction area of the 6th rib. **(C2, C3)** Postoperative bedside frontal and lateral chest X-rays showing partial absence of the left 6th rib, chest tube in the left pleural cavity, and soft tissue swelling with gas in the left chest wall as postoperative changes.

A 19-year-old Chinese female college student was found to have an abnormal chest lesion during a routine chest X-ray at university entry in August 2024 (**Figure 1C**). Further chest CT examination revealed bone destruction of the left 6th rib, accompanied by a soft tissue mass measuring approximately 68×119×93 mm. The lesion showed expansive and osteolytic changes, with ill-defined borders and patchy and arc-like calcifications, along with multiple septations. Enhanced CT images demonstrated heterogeneous enhancement of the mass, with destruction of the left 6th rib and adjacent soft tissue involvement (**Figures 1A, B**). The patient had no symptoms of chest pain, hemoptysis, dyspnea, fever, fatigue, or weight loss.

Physical examination revealed a well-oriented patient with stable vital signs and normal BMI. No palpable masses or lymphadenopathy were noted, and the respiratory, cardiovascular, and neurological systems showed no abnormalities.

**Figure 2:**
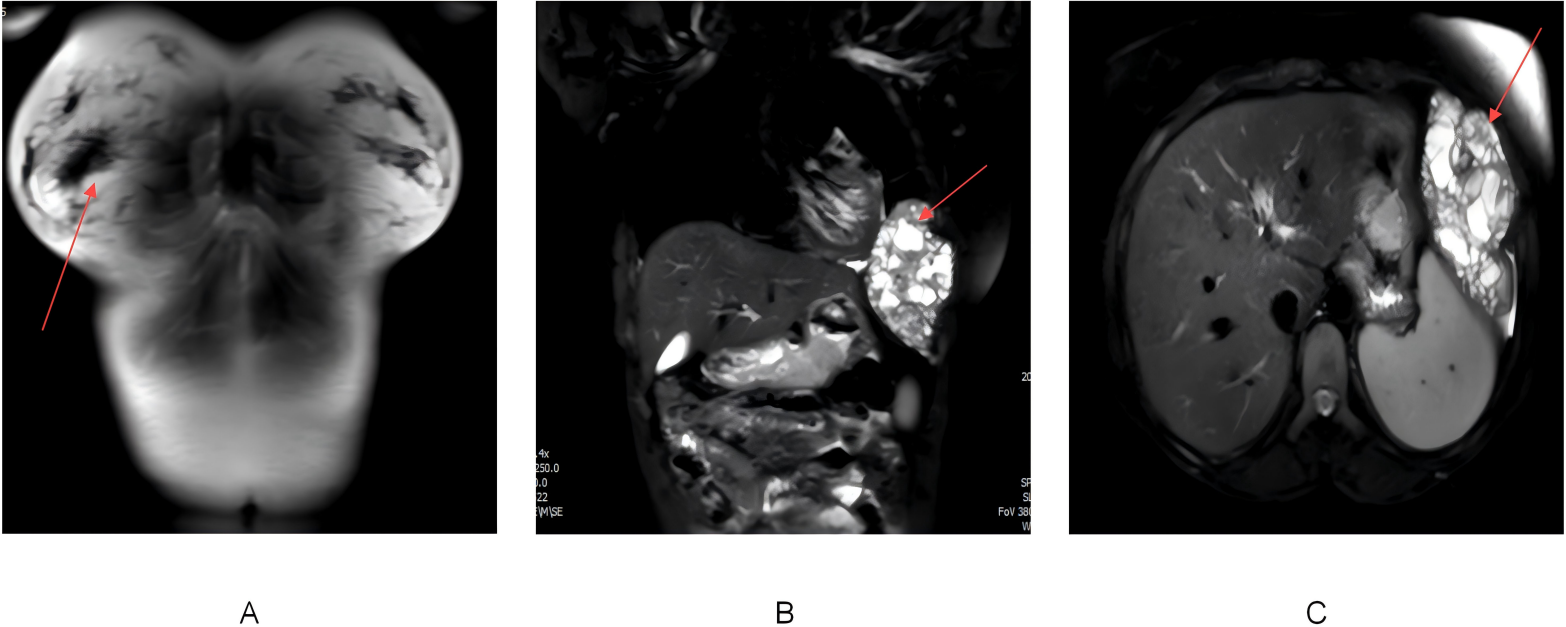
**(A)** MRI of the back and chest soft tissues showing expansile destruction of the left 6th rib, with an abnormal soft tissue mass in the adjacent pleural cavity. The mass appears heterogeneous on T1WI and T2-fat suppressed sequences. The red arrow marks the location of rib destruction and adjacent soft tissue mass. **(B)** Coronal MRI image showing a large soft tissue mass adjacent to the rib, with cystic degeneration and necrosis. The red arrow circles the cystic and necrotic area within the soft tissue mass. **(C)** Axial MRI image demonstrating the lesion compressing adjacent structures, including the left lung, diaphragm, and spleen. The red arrow points to the region of compression caused by the lesion.

Chest MRI further confirmed extensive bone destruction of the left 6th rib, along with a soft tissue mass measuring 116×67×102 mm. T1-weighted imaging showed slightly low signal intensity, while T2 fat-suppressed imaging displayed heterogeneous high signals. Coronal MRI images revealed cystic degeneration and necrosis within the mass, compressing adjacent structures such as the left lung and diaphragm (**Figures 2A–C**). Diffusion-weighted imaging (DWI) demonstrated areas of high signal intensity, and the apparent diffusion coefficient (ADC) map indicated decreased signal intensity, suggesting cystic changes and necrosis within the tumor.

**Figure 3:**
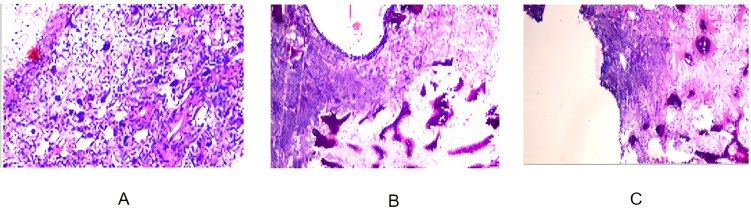
**(A)** Pathological section of the surgical specimen showing tumor tissue composed of spindle-shaped tumor cells and osteoclast-like giant cells, with significant cellular atypia and partial osteogenesis. **(B)** Pathological section showing a multi-cystic structure within the tumor tissue, with blood clots in the cysts and high mitotic activity, indicative of high-grade malignancy. **(C)** Pathological section showing cystic areas infiltrated by numerous spindle-shaped tumor cells and necrosis, consistent with features of a malignant giant cell tumor of bone.

On August 28, 2024, the patient underwent partial rib resection under general anesthesia at Lanzhou University Second Hospital. The resected tumor measured 14 × 10 × 7 cm, with a multiloculated cystic cut surface. Postoperative pathology revealed the tumor comprised spindle-shaped neoplastic cells and osteoclast-like giant cells, with significant cellular atypia and active mitoses (10/10 high-power fields), along with focal osteogenesis and cystic changes. Pathological examination confirmed the diagnosis of a malignant giant cell tumor (GCT) of the left 6th rib with chondrosarcomatous differentiation. Pathological sections demonstrated tumor tissues with prominent cellular atypia, cystic degeneration, and necrosis (**Figures 3A–C**). Immunohistochemistry results showed positivity for Vimentin, SMA, S-100, SATB2, Desmin, CD68, and CD163, while H3.3G34W, H3K36M, and CK were negative. The Ki-67 index ranged from 5% to 15%, indicating relatively high proliferative activity.

To confirm the diagnosis, the patient sought pathology consultation at Beijing Jishuitan Hospital on September 13 and at the First Medical Center of the PLA General Hospital on September 24. Both hospitals supported the diagnosis of malignant GCT with chondrosarcomatous differentiation. Immunohistochemical results at Jishuitan were consistent with those from Lanzhou University Second Hospital, while the PLA General Hospital showed positivity for H3.3G34W, p63, CD163, Vimentin, Desmin, and SMA, with a Ki-67 index of 25%.

The patient did not receive postoperative chemotherapy or radiotherapy. Follow-up evaluations began in October 2024, two months post-surgery, including imaging and physical examinations, which revealed no signs of recurrence. The patient recovered well without noticeable abnormalities and resumed her studies. A comprehensive follow-up strategy was developed, including imaging evaluations (CT or MRI) every three months to detect local recurrence or distant metastases. Monitoring tumor biomarkers was also included to assess disease progression and recurrence risks.

**Figure 4:**
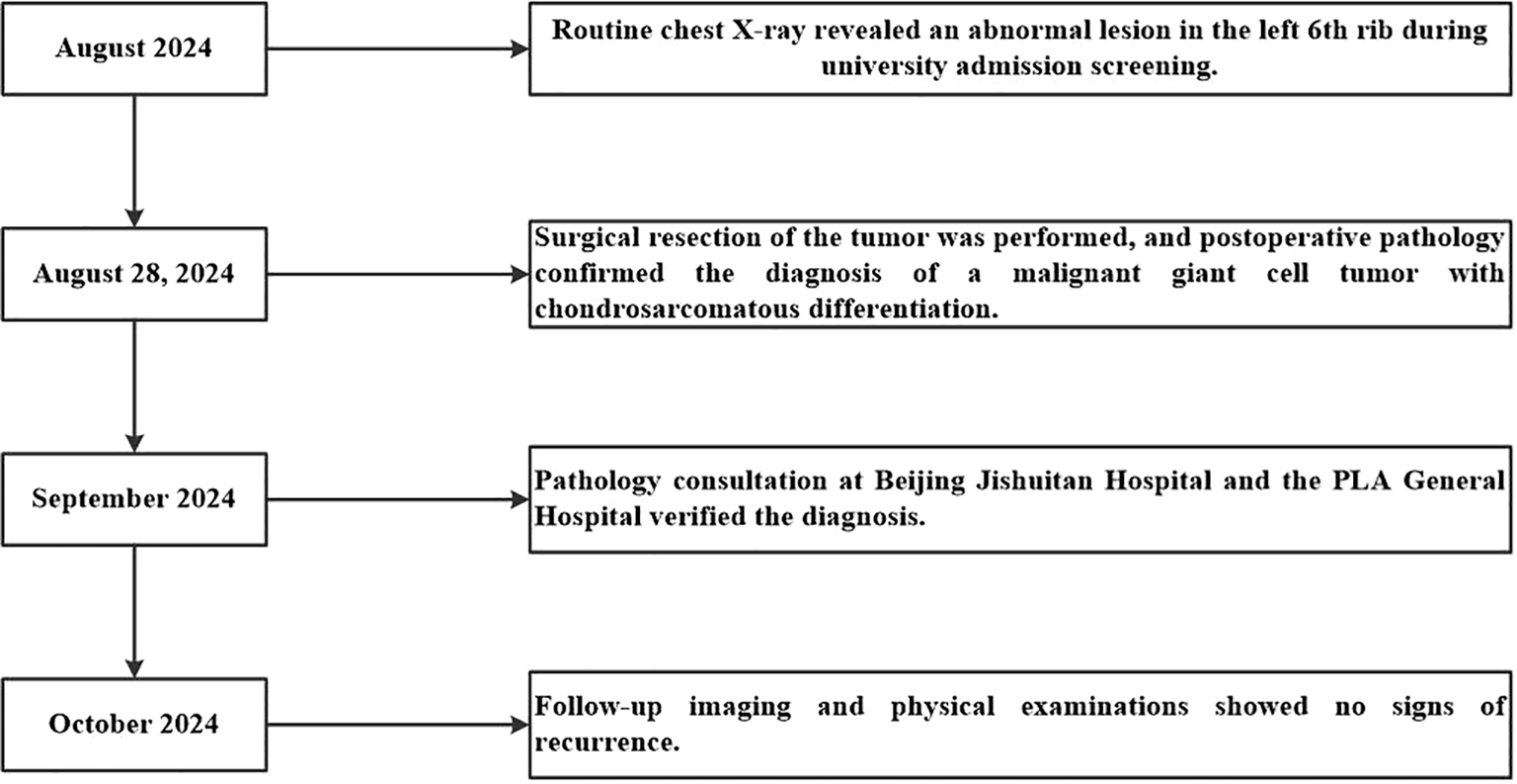
Timeline of key events in the diagnosis, treatment, and follow-up of the patient. This timeline summarizes the major clinical milestones for a 19-year-old female patient with a malignant giant cell tumor of the left 6th rib. Key events include the discovery of an abnormal chest lesion during routine university admission screening in August 2024, surgical resection and pathological confirmation of the diagnosis on August 28, 2024, pathology consultations in September 2024 that verified the diagnosis, and follow-up imaging and physical examinations in October 2024 showing no signs of recurrence.

To provide further context for this rare case, **Table 1** summarizes previously reported cases of Giant Cell Tumor (GCT) of the rib in adolescents. The table details patient demographics, clinical presentations, tumor characteristics, surgical approaches, pathological findings, and outcomes. This comparative summary highlights the variability in the presentation and management of rib GCTs, emphasizing the rarity of such cases and the necessity of individualized diagnostic and therapeutic strategies. A timeline summarizing key events, including diagnosis, treatment, and follow-up milestones, is presented in **Figure 4** to provide a clear chronological overview of the patient’s clinical course.

**Table 1:** Summary of reported cases of Giant Cell Tumor (GCT) of the rib in adolescents.

Case	Author & Year	Age (years)	Sex	Sign & Symptoms	Side and Location of GCT	Size (cm)	Surgery Approach	Pathology	Outcome
1	Locher GW et al., 1975	14	M	Pain	R, 6th rib, lateral arc	NA	En-bloc resection	GCT - ABC	NED, 3 years
2	Locher GW et al., 1975	13.5	M	Pain, mass	L, 8th rib, posterior arc	NA	En-bloc resection	GCT - ABC	NED, 5 years, scoliosis
3	Locher GW et al., 1975	4.5	F	No symptoms	R, 4th rib, posterior arc	NA	En-bloc resection*	GCT - ABC	NED, 1 year
4	Athanasiadou F et al., 2003	7	F	Mass	R, lower rib, posterior arc	8×4×4	Needle biopsy + En-bloc resection	GCT	NED, 1 year
5	Özyüksel G et al., 2020	12	F	Pain, mass	L, 7th rib, anterior arc	7×10×10	Tru-cut biopsy + En-bloc resection	GCT	NED, 14 months
6	Gui LS et al., 2009	21	M	Pain	Right, 9th rib	6×8×8	Tumor resection, bone cement filling	GCT - ABC	No recurrence, discharged
7	Huang Y et al., 2015	15	M	Pain	Left, 8th rib	NA	Curettage and bone grafting	GCT	Low recurrence rate (<12.9%)

M, male; F, female; NA, not available; R, right; L, left; *En-bloc resection for recurrent tumor previously underwent incomplete resection at another center, GCT, giant cell tumor; ABC, aneurysmal bone cyst; NED, no evidence of disease.

## 3 Discussion

This case of chondrosarcoma-like malignant giant cell tumor of the rib in an adolescent is representative of a highly aggressive bone tumor. Its rare location, occurrence in an adolescent, and chondrosarcomatous differentiation make both diagnosis and treatment extremely challenging. Multidisciplinary collaboration, particularly through a combination of imaging, pathology, and immunohistochemistry, helps improve diagnostic accuracy.

Chondrosarcomatous differentiation in GCT is rare and may involve complex cellular proliferation and tumor heterogeneity (11–13). Recent studies suggest that GCT formation may be associated with H3F3A gene mutations, which alter histone H3.3 function and affect cellular proliferation and differentiation (14, 15). H3F3A mutations are common in GCT patients, and identifying these mutations through genetic testing could not only reveal the tumor’s genetic characteristics but also provide scientific guidance for prognosis assessment and treatment strategy (14, 16). Additionally, abnormal expression of tumor suppressor genes like TP53, RB1, and MDM2 may play a role in malignant transformation during chondrosarcomatous differentiation, promoting tumor cell proliferation, inhibiting apoptosis, and increasing heterogeneity, thereby enhancing aggressiveness and recurrence risk (17).

Complete surgical resection remains the preferred treatment for chondrosarcoma-like malignant GCTs (18, 19). Despite successful resection, the tumor’s anatomical location and aggressive nature present technical challenges during surgery (15). If the tumor cannot be fully resected, radiotherapy or chemotherapy may be necessary to control tumor spread (18). With advances in molecular biology, targeted therapies for specific gene mutations, such as H3F3A or TP53, may improve patient outcomes in the future (14, 20). Recent studies suggest that combining bone-targeted therapies with tyrosine kinase inhibitors could provide novel therapeutic options for aggressive giant cell tumors, addressing both recurrence risks and tumor progression (21). These advancements highlight the importance of combining precise surgical techniques with emerging therapeutic strategies to address the aggressive behavior and recurrence potential of such tumors.

Although this patient showed no signs of recurrence within two months postoperatively, long-term monitoring remains crucial for managing these highly aggressive tumors (22). Regular follow-up should include periodic imaging studies, such as chest CT or MRI, to detect potential recurrence at the earliest stages (23, 24). Additionally, monitoring tumor biomarkers may provide supplementary information for assessing disease progression and recurrence risks (25). Given the high recurrence potential of chondrosarcoma-like malignant giant cell tumors, especially those with chondrosarcomatous differentiation, close surveillance is necessary to guide timely interventions and optimize long-term outcomes (26). A comprehensive follow-up strategy will be critical to ensure this patient’s continued recovery and to improve prognostic management for similar cases (27).

## 4 Conclusion

This case highlights the diagnostic and therapeutic challenges of managing chondrosarcoma-like malignant giant cell tumors of the rib in adolescents. The successful outcome through surgical treatment emphasizes the importance of multidisciplinary collaboration and individualized approaches for such rare tumors. Long-term follow-up remains essential to monitor recurrence risks and improve patient outcomes.

## Data Availability

The original contributions presented in the study are included in the article/supplementary material. Further inquiries can be directed to the corresponding author/s.
